# Determinants of social participation in patients living with systemic lupus erythematosus: the Psy-LUP multicentre study

**DOI:** 10.1136/rmdopen-2025-005661

**Published:** 2025-06-25

**Authors:** Cécile Manet, Marie-Anastasie Aim, Viviane Queyrel, Julien Faraut, Nathalie Costedoat-Chalumeau, Eric Daugas, Eric Hachulla, Jean-Robert Harle, Antoine Huart, Aurélie Hummel, Gilles Kaplanski, Karin Mazodier, Julien Mancini, Francoise Sarrot- Reynauld, Nicolas Schleinitz, Laure Swiader, Nathalie Tieulie, Philippe Manet, Lionel Dany, Laurent Chiche, Noemie Jourde-Chiche

**Affiliations:** 1 Service de Médecine Interne, CHU de la Conception, APHM, Marseille, France; 2 Délégation à la Recherche Clinique et à l’Innovation, AP-HM, Marseille, France; 3 LPS, Aix-Marseille Univ, Aix-en-Provence, France; 4 Internal Medicine, Universite Cote d’Azur, Nice, France; 5 Centre de Néphrologie et Transplantation Rénale, Marseille, France; 6 Internal Medicine Department, Referral center for rare autoimmune and systemic diseases, Assistance Publique-Hôpitaux de Paris (AP-HP), Cochin Hospital, Paris, France; 7 Descartes-Sorbonne Paris Cité University, Paris, France; 8 Université Paris Cité, Paris, France; 9 Nephrology, Hôpital Bichat - Claude-Bernard, Paris, France; 10 Lille University School of Medicine, Lille, France; 11 CHU Timone, Marseille, France; 12 Service de Néphrologie, Paris, France; 13 Internal Medicine and Clinical Immunology, Hôpital de la Conception, Marseille, France; 14 C2VN, Aix-Marseille University, Marseille, Provence-Alpes-Côte d’Azu, France; 15 Grenoble, Grenoble, France; 16 Service de Rhumatologie, Hôpital Pasteur, Centre Hospitalier Universitaire, Université de Nice Sophia Antipolis, Nice, France; 17 Revue Prescrire, Paris, France; 18 Aix-Marseille Universite, Marseille, France; 19 Internal Medicine, Hôpital Européen Marseille, Marseille, France; 20 C2VN, INSERM, INRAE, Aix-Marseille Univ, Marseille, France

**Keywords:** Lupus Erythematosus, Systemic, Social work, Qualitative research, Health-Related Quality Of Life

## Abstract

**Objective:**

Systemic lupus erythematosus (SLE) can negatively impact patients’ social participation. The aim of this study was to identify the determinants of social participation in patients with SLE.

**Methods:**

A cross-sectional evaluation was carried out in 100 adult outpatients with SLE enrolled in the multicentre psychosocial lupus (Psy-LUP) study. Participants completed the following standardised questionnaires: Participation Scale (social participation); Zimbardo Time Perspective Inventory; Sarason’s Social Support Questionnaire; Couples Satisfaction Index; Brief Illness Perceptions; Short Form-36 and Lupus-QoL. Stepwise multivariate regression analysis identified determinants of social participation.

**Results:**

92 women and eight men were included. Mean age was 44 years, mean SLE duration was 14 years, 52% of patients had a history of lupus nephritis and 38% were currently receiving immunosuppressants and/or biologics. 73% were in a couple and 64% were employed. Social participation was reduced in 29% of patients (compared with 46% in rheumatoid arthritis or multiple sclerosis), who reported different illness perceptions than those with preserved social participation. In multivariate linear regression, female sex (p=0.006), smoking (p=0.04), osteoporotic fractures (p=0.03), anti-cardiolipin antibodies (p=0.01) and ‘Past Negative’ time perspective (p=0.002) were associated with reduced social participation, while haematological involvement (p=0.005) and ‘Present Hedonistic’ time perspective (p=0.02) were protective. Reduced social participation was also associated with illness representations and with lower health-related quality of life (HR-QoL) scores.

**Conclusions:**

Social participation is frequently altered in patients with SLE and correlates with illness representations, time perspective and HR-QoL. Psychological support and therapeutic education may help improve patients’ time perspective.

**Trial registration number:**

NCT03913754.

**WHAT IS ALREADY KNOWN ON THIS TOPIC**

- Controlling disease activity is not enough to ensure optimal health-related quality of life (HR-QoL) of patients with systemic lupus erythematosus (SLE).
- Patients’ social participation has rarely been studied in SLE.

**WHAT THIS STUDY ADDS**

- Social participation is frequently impaired in patients with SLE.
- Altered social participation correlates not only with altered HR-QoL, but also with illness representations, and with time perspective, which refers to how individuals mentally view and relate to the past, present and future.

**HOW THIS STUDY MIGHT AFFECT RESEARCH, PRACTICE OR POLICY**

- Targeting modifiable determinants of social participation, through psychological support, cognitive therapy and therapeutic education, could help reduce the impact of SLE on patients’ daily life.

## Introduction

 Systemic lupus erythematosus (SLE) is a chronic systemic autoimmune disease, usually affecting women of childbearing age (sex ratio 9:1).^1^ Its global incidence and prevalence have increased worldwide over the last decades, but SLE remains a rare disease with around 30 000 prevalent cases in France.^2^ SLE can be severe and life-threatening or organ-threatening.^3 4^ Patients with SLE can be affected not only by inflammatory symptoms (eg, arthritis, mucocutaneous eruption, nephritis), known as ‘type 1’ symptoms and usually improved by immunosuppressive therapy, but also by ‘type 2’ symptoms (chronic fatigue, clinical depression, diffuse pain, sleep disorders, cognitive dysfunction), which are not associated with disease activity^5^ and can significantly alter physical and psychological quality of life.^6^

The disabling consequences of SLE symptoms, as well as the discrepancy between the concerns of physicians and patients, have led to a renewed interest in patient’s experience. By adopting a holistic, biopsychosocial approach,^7 8^ the therapeutic management of patients with SLE is directed towards improving their quality of life (QoL) and reducing the global impact of the disease rather than solely achieving clinical and biological remission.^5^ Social participation constitutes a useful concept for understanding patients’ daily life experiences. The assessment of social participation based on the International Classification of Functioning, Disability and Health (ICF) is widely used in chronic diseases.^9 10^ Social participation is defined by the ICF as “involvement in real-life situations”. It covers broad aspects of normal and community life, including the social, economic, civic, interpersonal, domestic and educational dimensions of daily living.^11^ Social participation restrictions are related to individual (disability, activity limitation, self-stigma), social (financial and material issues related to the disease, support and relationships) and societal (attitudes and systems, environment, policies, laws) factors. Social participation is closely linked to self-esteem, life satisfaction and mental health, making it a very important factor in health-related QoL (HR-QoL),^12^ particularly among people with disabilities.^13 14^

Restrictions on social participation have rarely been studied in patients with SLE. However, the fluctuating and unpredictable nature of SLE,^15^ pain, fatigue, organ damage, adverse effects of treatment and the need for sun protection can limit the ability of patients to maintain employment, leisure and family roles.^16^,^21^ Patients with SLE face body image and self-esteem issues,^22^ and the invisible and inconsistent nature of some symptoms can lead to a lack of understanding among family and caregivers, stigmatisation and social exclusion.^23^,^26^ The disease and its treatment can also interfere with parental plans.^27^,^30^

The primary objective of this study, part of the psychosocial lupus (Psy-LUP) research programme^21^ on the psychosocial consequences of SLE, was to identify the determinants of social participation in patients with SLE. The secondary objective was to investigate correlations between social participation and HR-QoL domains.

## Methods

### Participants and data collection

Between December 2018 and February 2020, this multicentre cross-sectional study included 100 adult outpatients with SLE according to Systemic Lupus International Collaborating Clinics (SLICC) criteria, followed up at eight centres in France.

The following standardised questionnaires were self-administered:

*Participation Scale (P-scale, PS)*,^31^ which assesses eight domains of social participation: learning and application of knowledge, communication, mobility, self-care, domestic life, interpersonal interactions and relationships, major life domains; and community, social and civic life. Respondents indicate whether they feel they have the same opportunities to participate in different life situations as their healthy peers, and, if not, the extent of the problem on a Likert scale. The higher the total score, the greater the restriction on social participation. Reduced social participation is defined by a total score of >12.

*Zimbardo Time Perspective Inventory (ZTPI)*,^32^ which measures time perspective in relation to the three temporal registers and people’s attitudes towards each of them. The 54-item ZTPI describes five subscales: ‘Past-negative’ (aversive attitude toward the past; for example, “It’s hard for me to forget unpleasant images of my youth”), ‘Past-positive’ (nostalgic, positive construction of the past; for example, “It gives me pleasure to think about my Past”), ‘Present-hedonistic’ (orientation toward enjoyment and pleasure in the present; for example, “Taking risks keeps my life from becoming boring”), ‘Present-fatalistic’ (hopeless, nihilistic attitude toward life; for example, “Since everything that will be, will be, what I do doesn’t really matter.”) and ‘Future’ (planning for and achievement of future goals; for example, “I complete projects on time by making steady progress”). These temporal frames are assessed on a 5-point Likert-type scale, ranging from 1 (very uncharacteristic) to 5 (very characteristic). For each individual, a time perspective profile made up of the scores for the five independent dimensions is compiled.

*Sarason’s Social Support Questionnaire (SSQ)*,^33^ which assesses the availability and satisfaction of social support.

*Couples Satisfaction Index (CSI)*,^34^ which provides a global score to assess the satisfaction of the couple relationship as perceived by the respondent.

*Brief Illness Perceptions Questionnaire (B-IPQ)*,^35^ which evaluates cognitive and emotional representations of the condition, as well as the individual’s understanding of its perceived causes. The higher the score, the more threatening the disease is perceived.

*HR-QoL* was assessed with the Short Form-36 (SF-36)^36 37^ and the SLE-specific questionnaire Lupus-QoL-FR,^38 39^ which comprises eight domains: physical health, pain, planning, fatigue, emotional health, body image, intimate relationships and burden to others.

Additional information on the questionnaires used is provided in online supplemental table S1.

###  Ethical approval

The Psy-LUP study was conducted with outpatients from Nephrology, Internal Medicine, and Rheumatology departments, as well as through telephone interviews conducted at home. The study had two main components: (1) the current quantitative phase, involving questionnaire-based data collection from SLE patients (n=100); and (2) a qualitative phase, consisting of research interviews with SLE patients (n=40)^21^ and their partners or spouses (n=20). The main objective of Psy-LUP was to assess the impact of lupus on patients’ lives, in terms of family life, relationships, social life and work.

Ethical approval for the Psy-LUP study (NCT03913754) was obtained from the Ile-de-France Committee for the Protection of Persons (reference number: A02747-48). All patients gave their written informed consent before they were given the questionnaires. At the same time, demographic and clinical data were collected. Data collected from partners were not analysed in this work.

### Statistical analysis

Demographic and clinical variables are reported as mean±SD or frequency. All parameters were tested for association with the P-scale score using Pearson’s linear correlation test (continuous parameters) or t-test (binary parameters). An additional analysis used the χ² or Fisher’s test to study the association between categorical parameters and the P-scale score divided into two groups: (i) ‘normal’ from 0‒12; and (ii) ‘reduced’ from 13‒100. The parameters of the same category were tested by multiple regression to select the most significant ones.

Sociodemographic and clinical parameters with a p value <0.1 in univariate analysis were included in a backward multiple linear regression analysis with the P-scale score as the dependent variable. The determinants selected at the end of this analysis were used to adjust the P-scale score to evaluate its association with other questionnaires (Lupus-QoL, SF-36 and B-IPQ). Stepwise multivariate regression analyses were then conducted to identify which dimensions of these questionnaires were significantly associated with the adjusted P-scale score. Each patient’s PS adjusted score was used to identify significant items in the various questionnaires by top-down multiple regression, adding the command ‘na.action’ = ‘na.omit’ to the ‘lm’ function in R software. All statistical analyses were performed using R version R 4.2.1 for MacOS.

## Results

### Study population

The characteristics of the 100 patients recruited are presented in table 1. There were 92 women and 8 men, with a mean age of 43 years. Patients were mainly of European (76%) or North African (16%) ancestry. The mean (SD) duration of SLE was 14±10 years, 52 (52%) patients had a history of lupus nephritis, five of whom had chronic kidney disease and one was on chronic dialysis. Immunosuppressive therapy, including biologic agents, was prescribed to 38% of patients, 35% were taking corticosteroids with a mean daily dose of 2.8 mg/day (15% at a dose >5 mg/day), and 75% were receiving hydroxychloroquine. Most participants (73%) were in couples, 65% had at least one child and 64% were employed.

**Table 1:** Sociodemographic and clinical characteristics of the study participants with systemic lupus erythematosus (SLE) and comparison between patients with normal (P-scale ≤12) or altered (P-scale >12) social participation (SP)

	All (n=100)	Normal SP (n=71)	Reduced SP (n=29)
Age (years)	44±14	43±14	47±13
Sex (female)	92 (92%)	63 (89%)	29 (100%)
Marital relationship	73/99 (73%)	56/70 (80%)	17 (59%)
Parent	65/99 (65%)	47/70 (67%)	18 (62%)
High school/higher degree	72/99 (73%)	49/69 (71%)	23 (79%)
Employment	63/99 (64%)	50/70 (71%)	13 (45%)
Active smoker	20 (20%)	10 (14%)	10 (34%)
SLE duration (years)	14±10	14±11	13±10
Number of SLE involvements	2.8±1.2	2.7±1.2	2.8±1.1
Cutaneous	89 (89%)	61 (86%)	28 (97%)
Articular	94 (94%)	65 (92%)	29 (100%)
Renal	52 (52%)	39 (55%)	13 (45%)
Haematological	41 (41%)	34 (48%)	7 (24%)
Cardiac	21/99 (21%)	14/70 (20%)	7 (24%)
Pulmonary	12 (12%)	10 (14%)	2 (7%)
Hepatic	6 (6%)	3 (4%)	3 (10%)
Neurological	9 (9%)	5 (7%)	4 (14%)
Other	5/98 (5%)	3 (4%)	2/27 (7%)
Antiphospholipid syndrome	20/99 (20%)	11/70 (16%)	9 (31%)
Lupus anticoagulant	24/99 (24%)	15/70 (21%)	9 (31%)
Anti-cardiolipin antibodies	18/99 (18%)	8/70 (11%)	10 (34%)
Anti-ß2GP1 antibodies	12/99 (12%)	5/70 (7%)	7 (24%)
High blood pressure	19 (19%)	14 (20%)	5 (17%)
Dyslipidaemia	11 (11%)	6 (8%)	5 (17%)
Diabetes	0 (0%)	0 (0%)	0 (0%)
Obesity	7 (7%)	4 (6%)	3 (10%)
Heart disease	8 (8%)	5 (7%)	3 (10%)
Osteoporosis	11/99 (11%)	7 (10%)	4/28 (14%)
Osteoporotic fracture	11/99 (11%)	5 (7%)	6/28 (21%)
Cataract	4 (4%)	2 (3%)	2 (7%)
Retinal damage	11/99 (11%)	6/70 (9%)	5 (17%)
Solid cancer	9 (9%)	7 (10%)	2 (7%)
Number of medications	3.7±2.4	3.4±2.3	4.6±2.7
Hydroxychroloquine	75 (75%)	53 (75%)	22 (76%)
Corticosteroids	35 (35%)	20 (28%)	15 (52%)
Corticosteroids >5 mg/day	15 (15%)	7 (10%)	8 (28%)
Corticosteroids ≥7.5 mg/day	12 (12%)	6 (8%)	6 (21%)
Corticosteroid daily dose (mg)	2.8±5.5	2.3±5.5	4.1±5.4
Immunosuppressant/biological	38 (38%)	24 (34%)	14 (48%)
Antidepressants	5 (5%)	1 (1%)	4 (14%)
Anxiolytics	3 (3%)	1 (1%)	2 (7%)
Anti-hypertensive/proteinuric	33 (33%)	25 (35%)	8 (28%)
Anti-osteoporotic	44 (44%)	35 (49%)	9 (31%)
Anti-coagulant/platelet agent	29 (29%)	16 (23%)	13 (45%)
Uric acid lowering	2 (2%)	1 (1%)	1 (3%)
Analgesics	10 (10%)	5 (7%)	5 (17%)
Cholesterol-lowering	4 (4%)	3 (4%)	1 (3%)
Contraceptive pill	10 (10%)	7 (10%)	3 (10%)
Thyroid hormones	13 (13%)	11 (15%)	2 (7%)
Anti-acid drugs	9 (9%)	5 (7%)	4 (14%)
Anti-pneumocystis prophylaxis	5 (5%)	2 (3%)	3 (10%)
Vitamins (B9, B12)	8 (8%)	5 (7%)	3 (10%)
Anti-asthma/allergy	5 (5%)	1 (1%)	4 (14%)
Eye drops	7 (7%)	4 (6%)	3 (10%)
Anti-epileptic	1 (1%)	0 (0%)	1 (3%)

Values are expressed as number (%) or as mean ± standard deviation. In the case of missing data, the number of available data is specified.

P-scale, Participation Scale.

### Social participation

**Figure 1:**
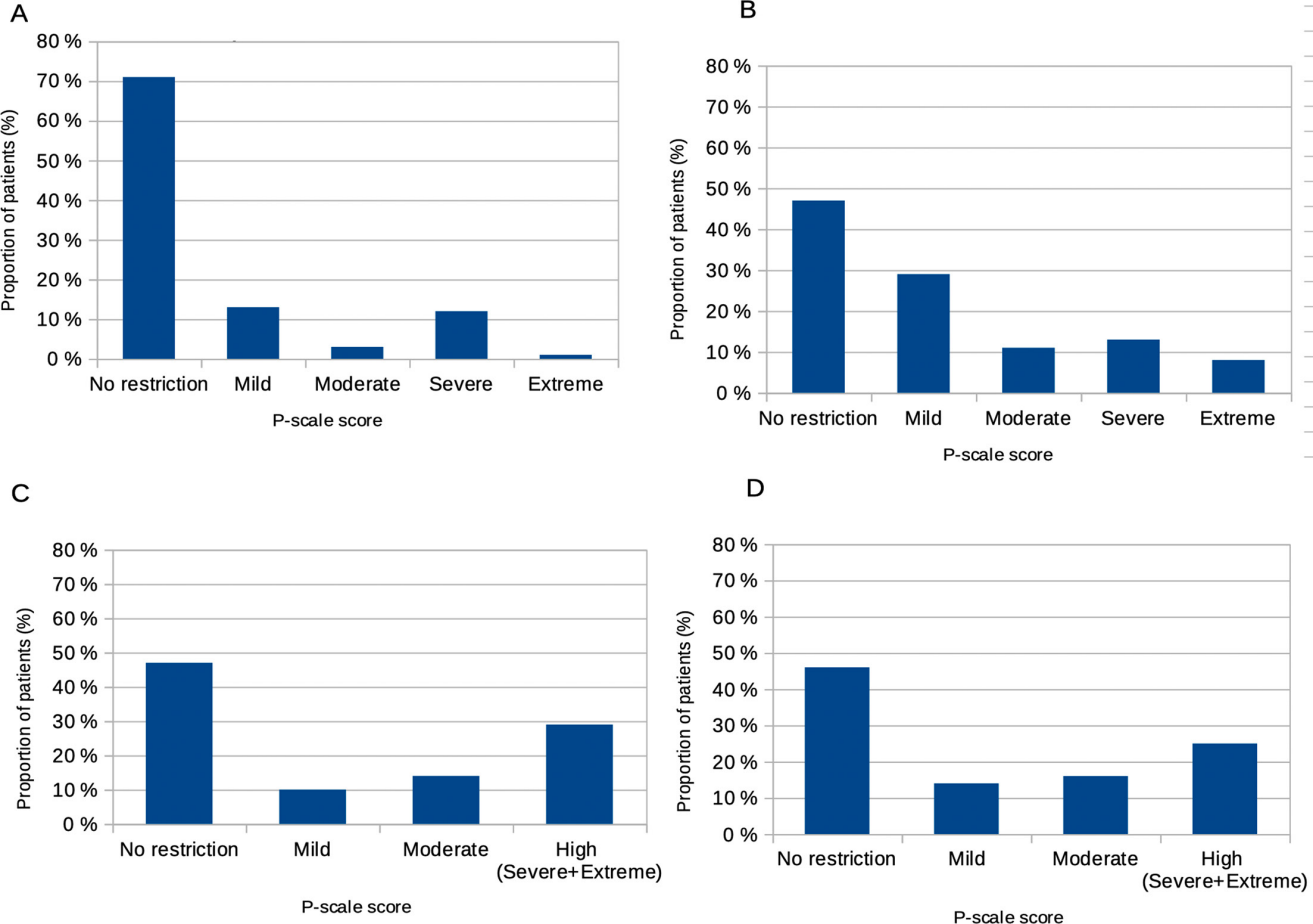
Social participation restrictions, evaluated by the P-scale in different populations. (0–12 no restriction; 13–22 mild; 23–32 moderate; 33–52 severe; and >52 extreme restriction). (**A**) Patients with systemic lupus erythematosus (SLE) from the Psy-LUP study. (**B**) Patients with multiple sclerosis from the study by Mikula *et al.*^36^ (**C, D**) Patients with rheumatoid arthritis (RA), early (**C**) or established (**D**), from the study by Benka *et al*.^37^ P-scale, Participation Scale; Psy-LUP, psychosocial lupus.

Social participation was altered in 29 patients (29% (95% CI: 20.4 to 38.9)) (P-scale score >12), 13 of whom had severe restriction of social participation (P-scale score >32). The distribution of social participation restriction categories is shown in figure 1 and compared with other autoimmune diseases such as multiple sclerosis^40^ or rheumatoid arthritis,^41^ in which social participation is again more frequently (46%) and more severely impaired.

### Psychosocial scores

Psychosocial scores are presented in online supplemental table S2. Couple satisfaction was better in patients with normal social participation but was assessed in only 53 patients and was thus not considered in the multivariate analysis. Patients with reduced social participation had a greater perception of the psychological burden of the illness (B-IPQ), perceived as more threatening and more often of external origin. Time perspective (ZTPI) also had an impact, with the ‘Past Negative’ perspective strongly associated with lower social participation.

### HR-QoL

**Figure 2:**
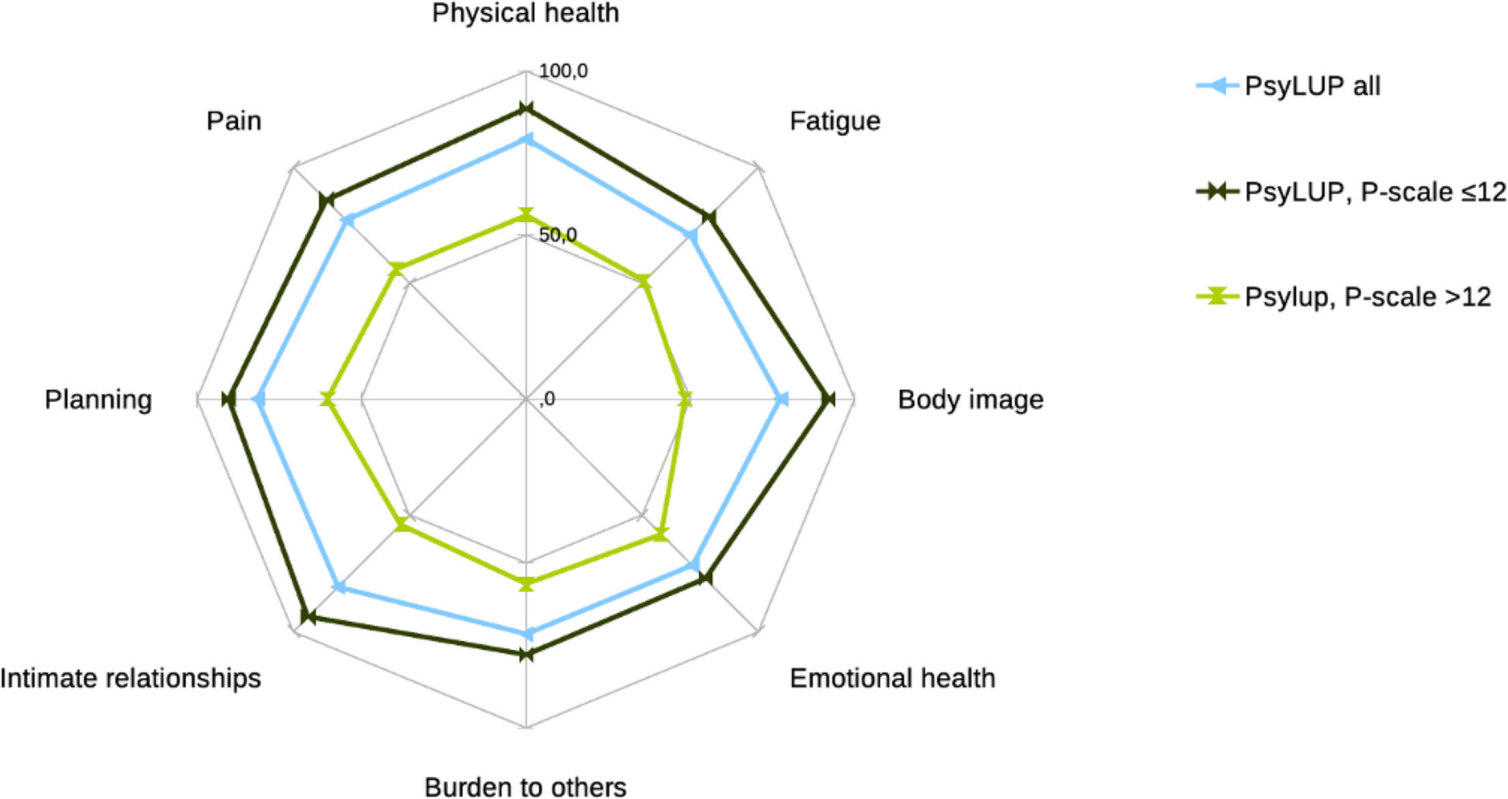
Spidergraph of Lupus-QoL domain scores for patients with systemic lupus erythematosus (SLE) in the psychosocial lupus (Psy-LUP) study. Values are shown for all Psy-LUP patients and for patients with normal (P-scale ≤12) or reduced (P-scale >12) social participation. The axes of the graph represent the different Lupus-QoL domains, graduated from 0 in the centre (worst) to 100 (best). Points represent the mean score of patients from a given cohort in each domain. Lines connect the scores of patients from a same cohort. P-scale, Participation Scale.

The results of the Lupus-QoL and SF-36 questionnaires are presented in online supplemental table S2. All scores were lower in patients with reduced social participation than in those with preserved social participation. Scatterplots of correlations between social participation and HR-QoL domains are presented in online supplemental figure S1. The Lupus-QoL domain scores of Psy-LUP patients as a function of social participation are shown in figure 2. In addition to the correlation between HR-QoL and social participation, the correlation also applied to all HR-QoL domains. These results were also compared with those of other cohorts of SLE patient in France,^38 42^ the USA^41^ and the UK^39^ (online supplemental figure S2).

### Determinants of social participation

In univariate analysis, patients with impaired social participation were more often unemployed (p=0.012), single (p=0.028), active smokers (p=0.021), had osteoporotic fractures (p=0.040) and anti-cardiolipin antibodies. They took more medications (p=0.026), more frequently corticosteroids (p=0.025), especially at doses >5 mg/day (p=0.033), antidepressants (p=0.024), anti-platelet agents (p=0.026) and anti-asthmatics (p=0.024). They had less haematological involvement (p=0.028).

Multivariate stepwise regression analysis (table 2) identified seven independent predictors of social participation: haematological involvement (p<0.01) and ‘Present Hedonistic’ time perspective (p=0.02) were protective, while ‘Past Negative’ time perspective (p=0.002), female gender (p=0.006), smoking (p=0.04), osteoporotic fracture (p<0.05) and anti-cardiolipin antibodies (p=0.015) were predictive of lower social participation.

**Table 2:** Multivariate (stepwise linear regression) analysis of factors predictive of the P-scale score

Predictive factor	β	SE	T	P value
ZTPI: Past Negative	3.8	1.2	3.234	0.002**
ZTPI: Present Hedonistic	−3.5	1.5	−2.366	0.020*
Haematological disorders	−6.9	2.4	−2.891	0.005**
Sex (female)	12.2	4.3	2.823	0.006**
Active smoker	6.7	3.3	2.073	0.041*
Osteoporotic fracture	8.5	4.0	2.136	0.035*
Anti-cardiolipin antibodies	8.1	3.3	2.479	0.015*

* p<0.05, **p<0.01.

P-scale, Participation Scale; T, Student coefficient; ZTPI, Zimbardo Time Perspective Inventory; β, estimated regression coefficient.

Among the Lupus-QoL and SF-36 domains and disease representations (B-IPQ), we also analysed which ones were the strongest predictors of social participation, using a stepwise regression analysis adjusted for demographic and clinical factors. The results are shown in online supplemental table S3. The Lupus-QoL domains most strongly linked to social participation were physical health (p=0.001) and planning (p<0.05). In the SF-36, physical functioning (p<0.001) and vitality (p=0.01) showed the strongest associations. For the B-IPQ, the ‘Consequences’ domain (p<0.001), which reflects the perceived impact of illness on life (with greater perceived impact linked to lower social participation), and ‘Personal Control’ (p=0.02), where a stronger sense of control was associated with better social participation, were the most relevant.

## Discussion

The results of this multicentre study show that social participation is impaired in almost one-third of patients living with SLE and is strongly associated with reduced HR-QoL. In addition to demographic parameters (female sex), disease and treatment-related parameters (anti-cardiolipin antibodies, haematological involvement or osteoporotic fractures), altered time perspective and smoking were independent predictors of social participation.

Not all organ involvements in SLE have an equal impact on social participation. A history of haematological involvement, one of the ‘invisible’ aspects of the disease, was more frequent in patients with normal social participation. This is consistent with previous reports of a more pronounced HR-QoL alteration in patients with cutaneous-articular than with haematological SLE manifestations.^42^ Interestingly, as suggested in a recent study from our group,^21^ no association was found between a history of lupus nephritis or kidney damage and social participation (although only one patient was on dialysis), which highlights the discrepancy between physicians’ concerns (severity of SLE in the case of lupus nephritis) and impact on patients’ daily life.^43 44^ The fact that anti-cardiolipin antibodies were predictive of reduced social participation could be related to an increased risk of thrombotic events (eg, stroke, deep vein thrombosis) leading to physical disability and mobility restrictions, to long-term anticoagulation therapy, which introduces bleeding risks that may hinder social engagement, or to more severe disease phenotype, as anti-phospholipid antibody positivity is associated with higher cardiovascular risk, lupus nephritis and chronic illness burden. This interesting finding should be validated and analysed in greater detail in future studies.

In the context of chronic diseases, the process of chronicity raises specific questions about how the perception of time influences emotions, behaviours and personal experiences. The concept of time perspective (TP) provides a relevant conceptual framework for thinking and operationalising (ie, allowing to measure) the individual and the social experience of the relationship to time. Time perspective is the often non-conscious process whereby the continual flows of personal and social experiences are assigned to temporal categories, or time frames, that help to give order, coherence and meaning to those events.^32^ It refers to the dimension of the psychological construction of time and can be defined as the relationship that individuals and groups have with the present, past and future. In a prospective cohort of patients with SLE, time perspective evaluated by ZTPI was an independent predictor of SLICC damage.^45^ Future-oriented patients were less likely to have an increased damage index than present-oriented patients. In our study, patients with poorer social participation had altered time perspective, with higher scores in the ‘Past Negative’ dimension (pessimistic, negative or aversive attitude towards the past) and ‘Present Fatalistic’ dimension (resignation, lack of control over future). Conversely, ‘Present Hedonistic’ (quest of present pleasure with little concern for future) was protective for social participation. Time perspective could therefore also affect coping strategies and HR-QoL, as shown in other contexts.^46^,^49^ The mental capacity to switch between time perspectives^32^ is a sort of temporal plasticity which allows individuals to maximise their adaptation to changing environmental conditions and to effectively realise their personal goals and values. Inspired by narrative therapy approach, the Time Perspective Therapy^50^ was developed to improve the patient’s ability to believe in themselves, in their efficacy to move forward in life, rather than rehashing the negative past. Mindfulness-based interventions can also constitute a useful way to develop a more balanced time perspective.^51^

To date, little data are available on social participation in patients living with SLE. In our cohort, social participation was less altered than in patients living with multiple sclerosis^40^ or rheumatoid arthritis.^41^ This could be partly explained by some haematological or renal SLE manifestations that are mildly symptomatic regardless of their severity. Moreover, our cohort was mainly composed of quiescent outpatients. Mikula *et al* used the P-scale in multiple sclerosis^52^ and reported that social participation was significantly associated with disability and disease duration, and with the physical component summary of the SF-36 after controlling for sociodemographic and clinical variables, while it was not associated with the mental component summary of the SF-36. Benka *et al* used the P-scale in patients with rheumatoid arthritis^53^ and found that the level of social participation restriction correlated with some clinical characteristics (pain, fatigue and functional disability) but also with psychological status (anxiety, depression) and personal resources (self-esteem, mastery). These discrepancies suggest that depending on the disease, social participation restrictions may be more or less mediated either by physical disability or by psychosocial factors. Future research deepening the understanding of such specifics and exploring the expectations of patients with diverse autoimmune diseases regarding standard of participation would be insightful for both rehabilitation clinicians and researchers. Cano-Garcia *et al* studied the association of different factors with social participation, assessed with the PROMIS-APS score, in 151 patients with inflammatory rheumatic diseases (rheumatoid arthritis, spondylarthropathy and SLE).^54^ Social participation was not associated with disease activity but was inversely related to depression and directly with social satisfaction, mobility, company and age. A systematic review of 14 articles^55^ identified that the presence of cognitive impairment is very common in SLE patients (prevalence of 38%) and is negatively related to HR-QoL and social participation. It would have been interesting to measure cognitive function in our study, especially to explore the link with depression, which seemed to be more frequent in patients with reduced social participation since they took more antidepressants. A longitudinal cohort study examined the coping processes in SLE patients that lead to improved HR-QoL.^56^ This study highlighted the detrimental role of impaired social participation and depression, and the favourable role of intrinsic factors (good mental health status, self-efficacy feeling). Surprisingly, no association was found in this study between coping scores and fatigue or physical activity.

Our study has some limitations: (i) selection bias is possible, since participants were outpatients with a long history of SLE, recruited on a voluntary basis by lupus specialists in nephrology and internal medicine or rheumatology (none from dermatology); of note, the refusal rate for participation was zero which may account for a great interest of our patients for a research about these less studied aspects of the disease; (ii) SLE disease activity score (such as SLEDAI) was not assessed at the time of the visit for Psy-LUP (no blood sample was drawn in this non-interventional study). Yet, the disease was generally clinically quiescent (only 15 patients took >5 mg/day prednisolone, disqualifying them from being in remission according to the DORIS definition^57^) and previous study including patients with SLE found no association between social participation and disease activity^54^; regarding evaluation of damages, SLICC Damage Index was not used either, but main items were collected and analysed (table 1); (iii) precarity and socioeconomic status, which are known to be a confounding factor for QoL^58^ and social participation, were not assessed, as well as additional objective cognitive self-assessments (eg, PHQ-9, HADS) that should be included in future studies;^55^ (iv) due to the cross-sectional study design, causality cannot be proven. Future longitudinal studies could be carried out to explore the causal links between social participation and predictive factors; (v) many responses were missing in the domains intimate relationships and body image of the Lupus-QoL, which could not always be scored. This limit has been raised before in the French validation study of Lupus-QoL^38^; in addition, too much data were missing for CSI and SSQ questionnaires, and thus, were also omitted from multivariate analyses. However, once controlled, they were little associated with P-scale in the exploratory analyses; (vi) the P-scale used here to evaluate social participation has been criticised for including dimensions that are more about autonomy than social participation;^59^ however, it is built on the ICF definition that could also be criticised in the way in which participation may not sufficiently address social involvement;^60^ (vii) the P-scale invite participants to compare themselves to someone who would be similar to them in every aspect except for the disease. The research results provide information on the subjective discrepancy between the social participation of patients with SLE and healthy peers. However, no data on social participation using the P-scale are available on age and sex-matched healthy individuals, preventing to provide a clearer measure for social participation in a healthy control group. Future research should be led in order to study how much of the observed impairment accounts to SLE itself against other psychosocial factors; (viii) several criticisms have been levelled against the ICF definition of social participation,^60^ mostly regarding the need to both clarify and extend its definition, and to address further structural and sociocultural involvement. Although the authors tend to agree with deepen the ICF’s conceptualisation, no definition of social participation seems to stand out in the current conceptual debate. Considering the wide use of terminology and classifications as well as participation assessments based on the ICF in chronic diseases,^9 10^ and that few data are available on social participation in patients with SLE, especially in the French context, we choose to use a social participation assessment tool (the Participation Scale) based on the ICF definition to facilitate the linking of our results with the existing literature as well as comparisons with other populations with autoimmune diseases. Although the clarification of the definition of social participation is beyond the scope of this research, the assessment of social participation in SLE using a diversity of tools based on various conceptualisation of participation constitutes a fruitful research perspective that would benefit both clinical domain and theorical and methodological development.

This study also has many strengths. It is the first study to use peer comparisons to measure the social participation of patients living with SLE. This strategy emphasises the patients’ perspectives on the impact of the disease on their lives. The study contributes to improve our knowledge on SLE since it provides original data from seven psychosocial questionnaires in a population of SLE patients. Two of them (P-scale and CSI) had never been used in SLE and ZTPI has only been used once.^45^ It opens the field for future psychosocial studies in SLE. The study size was relatively important for a rare disease, with detailed clinical and sociodemographic data.

In conclusion, social participation is frequently altered in patients with SLE and correlates with altered HR-QoL, illness representations and time perspective. The Psy-LUP study is an important step to understanding how SLE impacts patients’ ability to maintain their social roles. Controlling disease activity is not enough to ensure optimal HR-QoL. Supporting social participation of patients is crucial to reduce the impact of the disease on their daily life. Psychological support, cognitive therapy and therapeutic education, among others, should be widely offered to support patients’ coping processes, particularly by improving their sense of self-efficacy and possibly by modifying their time perspective. Future studies are needed to evaluate the impact of such interventions on patients’ HR-QoL.

## Supplementary material

10.1136/rmdopen-2025-005661 online supplemental file 1

## Data Availability

All data relevant to the study are included in the article or uploaded as supplementary information.
