# Supplementary material for: Determinants of social participation in patients living with systemic lupus erythematosus: the Psy-LUP multicentre study
Source: RMD Open. 2025 Jun 25;11(2):e005661. doi: 10.1136/rmdopen-2025-005661 (PMC12198841; doi:10.1136/rmdopen-2025-005661)
Supplement: online supplemental file 1 [file rmdopen-11-2-s001.docx]

# SUPPLEMENTARY MATERIAL

**Table S1: Information about questionnaires**

| **Questionnaires/scales** | **Description** | **Number of domains/Items** | **Cut offs** |
| --- | --- | --- | --- |
| **P-scale**  **Participation Scale** | Assesses of social participation (person’s involvement in a life situation) | 18 items covering eight of the nine major life domains (learning and applying knowledge, communication, mobility, self-care, domestic life,  interpersonal interactions and relationships, major life  areas, and community, social and civic life) | Reduced social participation is defined by a total score of >12 |
| ***ZTPI***  **Zimbardo Time Perspective Inventory** | Assesses how individuals mentally view and relate to the past, present, and future | 5 domains (past-positive; past-negative; present-hedonistic; present-fatalistic; future) | NA |
| ***SSQ***  **Social Support Questionnaire** | Measures dimensions of social support | 2 dimensions (satisfaction and availability) | NA |
| ***CSI***  **Couple Satisfaction Index** | assesses the satisfaction of the couple relationship as perceived by the respondent | 32 items (global score) | NA |
| ***B-IPQ****  **Brief Illness Perceptions Questionnaire** | evaluation of cognitive and emotional representations of the illness and illness understanding | 9 items, 5 assess cognitive perceptions (consequences, timeline, personal control; treatment control, identity); 3 assess emotional representation (illness concern, understanding, emotional response); 1 asking respondents to rank the three most important factors causing their illness | NA** |
| **SF-36**  **Qol** | Measure functional health and well-being from the patient’s (generic measure) | 8 domains (physical functioning, social functioning, role physical, role emotional, mental health, energy/vitality, pain, general health) | NA |
| **Lupus-QoL-FR** | Assesses QoL for SLE patients (specific measure) | 8 domains (physical health, pain, planning, fatigue, emotional health, body image, intimate relationships and burden to others) | NA |

* The psychosocial questionnaires used in this study are time-consuming, but the B-IPQ regression analysis showed that the item Consequences ("How does your illness affect your life?" scored 0‒10) is a good predictor of social participation and could be asked routinely in consultation to identify patients requiring further assessment and support for their social participation. ** The higher the score, the more threatening the disease is perceived.

**Table S2:** **Psychosocial scores for the patients with systemic lupus erythematosus** **and comparison between patients with normal (P-scale ≤12) or reduced (P-scale >12) social participation (SP).**

|  | **All**  **(n=100)** | | **Normal SP**  **(n=71)** | | **Reduced SP**  **(n=29)** | | **R2** | **p value** |  |  |  |  |  |  |
| --- | --- | --- | --- | --- | --- | --- | --- | --- | --- | --- | --- | --- | --- | --- |
| **Lupus-QoL** | n |  | n |  | n |  |  |  |  |  |  |  |  |  |
| Physical Health | 99 | 79.1 ± 22.1 | 70 | 88.6 ± 12.7 | 29 | 56 ± 23.1 | 0.61 | 1.1E-21 *** |  |  |  |  |  |  |
| Pain | 99 | 77.1 ± 24.9 | 70 | 85.8 ± 17.5 | 29 | 56 ± 27.6 | 0.42 | 5.8E-13 *** |  |  |  |  |  |  |
| Planning | 99 | 81.6 ± 25.1 | 70 | 90.5 ± 17.5 | 29 | 60.3 ± 28.2 | 0.46 | 1.2E-14*** |  |  |  |  |  |  |
| Intimate Relationship | 71 | 80.8 ± 28.6 | 48 | 93.8 ± 12.1 | 23 | 53.8 ± 34.2 | 0.39 | 5.5E-09*** |  |  |  |  |  |  |
| Burden to Others | 99 | 71.5 ± 25.3 | 70 | 77.9 ± 21.9 | 29 | 56.3 ± 27 | 0.21 | 1.6E-06*** |  |  |  |  |  |  |
| Emotional Health | 99 | 71.6 ± 20 | 70 | 77.1 ± 16.7 | 29 | 58.2 ± 21.1 | 0.32 | 9.2E-10*** |  |  |  |  |  |  |
| Body Image | 36 | 77.4 ± 26.9 | 24 | 91.9 ± 12.4 | 12 | 48.3 ± 24.4 | 0.41 | 9.3E-10*** |  |  |  |  |  |  |
| Fatigue | 100 | 70.6 ± 24.4 | 71 | 78.7 ± 19.6 | 29 | 50.9 ± 23.8 | 0.39 | 2.6E-12*** |  |  |  |  |  |  |
| **SF-36** |  |  |  |  |  |  |  |  |  |  |  |  |  |  |
| Physical Functioning | 100 | 76.6 ± 25.9 | 71 | 85.4 ± 20.7 | 29 | 55 ± 24.8 | 0.46 | 1.1E-14*** |  |  |  |  |  |  |
| Social Functioning | 100 | 69.9 ± 23.2 | 71 | 77.5 ± 19 | 29 | 51.3 ± 22.5 | 0.37 | 2.1E-11*** |  |  |  |  |  |  |
| Role Physical | 100 | 65.2 ± 41 | 71 | 76.8 ± 34.2 | 29 | 36.8 ± 42.9 | 0.29 | 5.7E-09*** |  |  |  |  |  |  |
| Role Emotional | 100 | 67 ± 40.1 | 71 | 81.2 ± 30.2 | 29 | 32.2 ± 40.3 | 0.29 | 7.3E-09*** |  |  |  |  |  |  |
| Mental Health | 100 | 62.2 ± 18.7 | 71 | 68.2 ± 15.3 | 29 | 47.7 ± 18.5 | 0.26 | 6.9E-08*** |  |  |  |  |  |  |
| Energy/vitality | 100 | 49.4 ± 20.2 | 71 | 55.9 ± 18 | 29 | 33.3 ± 15.9 | 0.29 | 5.8E-09*** |  |  |  |  |  |  |
| Bodily Pain | 100 | 54.9 ± 9 | 71 | 56 ± 8.6 | 29 | 52.3 ± 9.6 | 0.07 | 0.008*** |  |  |  |  |  |  |
| General Health | 100 | 53.1 ± 24 | 71 | 61.3 ± 20.7 | 29 | 32.9 ± 19.2 | 0.37 | 2.3E-11*** |  |  |  |  |  |  |
| MCS | 100 | 44.4 ± 8.3 | 71 | 46.7 ± 6.8 | 29 | 38.8 ± 8.8 | 0.36 | 5.8E-11*** |  |  |  |  |  |  |
| PCS | 100 | 44.1 ± 11.1 | 71 | 47.8 ± 8.9 | 29 | 35.1 ± 10.9 | 0.25 | 1.2E-07*** |  |  |  |  |  |  |
| **Couple Satisfaction Index (CSI) (a)** | | |  |  |  |  |  |  |  |  |  |  |  |  |
| CSI | 53 | 61.8 ± 19.6 | 39 | 66.4 ± 15.1 | 14 | 49.2 ± 25.2 | 0.12 | 0.013** |  |  |  |  |  |  |
| **Brief Illness Perceptions Questionnaire (B-IPQ)** | | | | | | | | |  |  |  |  |  |  |
| Consequences | 100 | 4.8 ± 2.9 | 71 | 3.8 ± 2.5 | 29 | 7.2 ± 2.2 | 0.42 | 3.1E-13*** |  |  |  |  |  |  |
| Timeline | 100 | 8.6 ± 2.5 | 71 | 8.3 ± 2.7 | 29 | 9.3 ± 2 | 0.06 | 0.018** |  |  |  |  |  |  |
| Personal Control | 100 | 5.9 ± 2.7 | 71 | 6.2 ± 2.6 | 29 | 5.1 ± 2.9 | 0.04 | 0.042* |  |  |  |  |  |  |
| Treatment Control | 99 | 8 ± 2.4 | 70 | 8 ± 2.6 | 29 | 7.9 ± 2 | 0.00 | 0.965 |  |  |  |  |  |  |
| Identity | 100 | 4.3 ± 2.8 | 71 | 3.5 ± 2.7 | 29 | 6.2 ± 2.1 | 0.23 | 3.7E-07*** |  |  |  |  |  |  |
| Illness Concern | 100 | 7.6 ± 2.8 | 71 | 7.2 ± 3.1 | 29 | 8.7 ± 1.8 | 0.07 | 0.009*** |  |  |  |  |  |  |
| Understanding | 100 | 7.8 ± 2 | 71 | 7.6 ± 2.1 | 29 | 8.2 ± 1.7 | 0.01 | 0.413 |  |  |  |  |  |  |
| Emotional Response | 99 | 5.9 ± 3 | 71 | 5.1 ± 2.9 | 28 | 7.8 ± 2.4 | 0.26 | 7.0E-08*** |  |  |  |  |  |  |
| External Causality | 100 | 17 (17%) | 71 | 7 (10%) | 29 | 10 (34%) | 0.00 | 0.006*** |  |  |  |  |  |  |
| **Sarason’s Social Support Questionnaire (SSQ)** | | | | | | | | |  |  |  |  |  |  |
| Availability | 80 | 21 ± 9.1 | 62 | 20.8 ± 9.5 | 18 | 21.8 ± 7.5 | 0.00 | 0.95 |  |  |  |  |  |  |
| Satisfaction | 74 | 28 ± 10 | 59 | 27.8 ± 10.2 | 15 | 28.7 ± 9.3 | 0.02 | 0.18 |  |  |  |  |  |  |
| **Zimbardo Time Perspective Inventory (ZTPI)** | | | | | | | | |  |  |  |  |  |  |
| Past Negative | 99 | 3.1 ± 1 | 71 | 3 ± 1 | 28 | 3.5 ± 1.1 | 0.12 | 0.0004*** |  |  |  |  |  |  |
| Present Hedonistic | 98 | 3.2 ± 0.8 | 70 | 3.3 ± 0.7 | 28 | 3.1 ± 1 | 0.02 | 0.125 |  |  |  |  |  |  |
| Future | 100 | 3.4 ± 0.5 | 71 | 3.4 ± 0.5 | 29 | 3.5 ± 0.5 | 0.01 | 0.442 |  |  |  |  |  |  |
| Past Positive | 97 | 3.7 ± 0.7 | 69 | 3.7 ± 0.8 | 28 | 3.8 ± 0.6 | 0.00 | 0.800 |  |  |  |  |  |  |
| Present Fatalistic | 100 | 2.2 ± 0.9 | 71 | 2.2 ± 0.9 | 29 | 2.3 ± 0.9 | 0.05 | 0.032* |  |  |  |  |  |  |

Values are expressed as mean ± standard deviation or n (%). The coefficient of linear correlation (R2) of each score with P-scale linear value is shown. The number of available data is specified for each score.

MCS: mental component summary; PCS: physical component summary.

(a) Couple Satisfaction Index 16-item version, scored from 0 to 81 : The score should be compared with data for a couple of the same duration, but a score < 51.5 usually indicates significant unsatisfaction. (29)

**Table S3: Stepwise regression analysis of Lupus-QoL, SF-36 and BIP-Q domains on the P-scale score.**

| **Lupus-QoL domain** | **β** | **SE** | **t** | **p value** |
| --- | --- | --- | --- | --- |
| (Intercept) | 29.10 | 4.10 | 7.10 | 1.01E-09*** |
| Physical Health | -0.27 | 0.08 | -3.40 | 0.00114** |
| Planning | -0.18 | 0.09 | -2.02 | 0.04727* |
| Intimate Relationship | 2.18 | 1.61 | 1.35 | 0.1808 |

Multiple R-squared 0.73

| **SF-36 domain** | **β** | **SE** | **t** | **p value** |
| --- | --- | --- | --- | --- |
| (Intercept) | 20.10 | 3.30 | 6.10 | 2.2E-08*** |
| Physical Functioning | -0.17 | 0.04 | -3.72 | 0.000337*** |
| Vitality | -0.15 | 0.06 | -2.61 | 0.010532* |

Multiple R-squared 0.58

| **SF-36 component** | **β** | **SE** | **t** | **p value** |
| --- | --- | --- | --- | --- |
| (Intercept) | 40.32 | 6.46 | 6.24 | 1.12E-08*** |
| PCS | -0.60 | 0.12 | -4.95 | 3.11E-06*** |
| MCS | -0.31 | 0.09 | -3.40 | 0.000992*** |

Multiple R-squared 0.59

| **BIP-Q** | **β** | **SE** | **t** | **P value** |
| --- | --- | --- | --- | --- |
| (Intercept) | -4.82 | 2.95 | -1.63 | 0.1053 |
| Consequences | 2.05 | 0.35 | 5.87 | 6.07E-08*** |
| Personal Control | -0.86 | 0.36 | -2.36 | 0.0201* |

Multiple R-squared 0.57

ß : estimated regression coefficient
SE : standard error

t : Student coefficient

Multiple R-squared : coefficient of determination (proportion of the variation in dependent variable that can be explained by the independent variables)

Each model was adjusted for demographic and clinical factors (sex, active smoking, osteoporotic fractures, haematological disorders, anti-cardiolipin antibodies, anti-osteoporotic treatment).

PCS: physical component summary; MCS: mental component summary.

**Figure S1:** **Correlations between social participation (P-scale score) and Quality of Life (Lupus-QoL domains (2a) or SF-36 domains or Mental (MCS) and Physical Component Summary (PCS) (2b).**

**Figure S2:** **Spidergraph of Lupus-QoL domains: comparison of the Psy-LUP population to other cohorts of patients with systemic lupus erythematosus from the literature.**

**
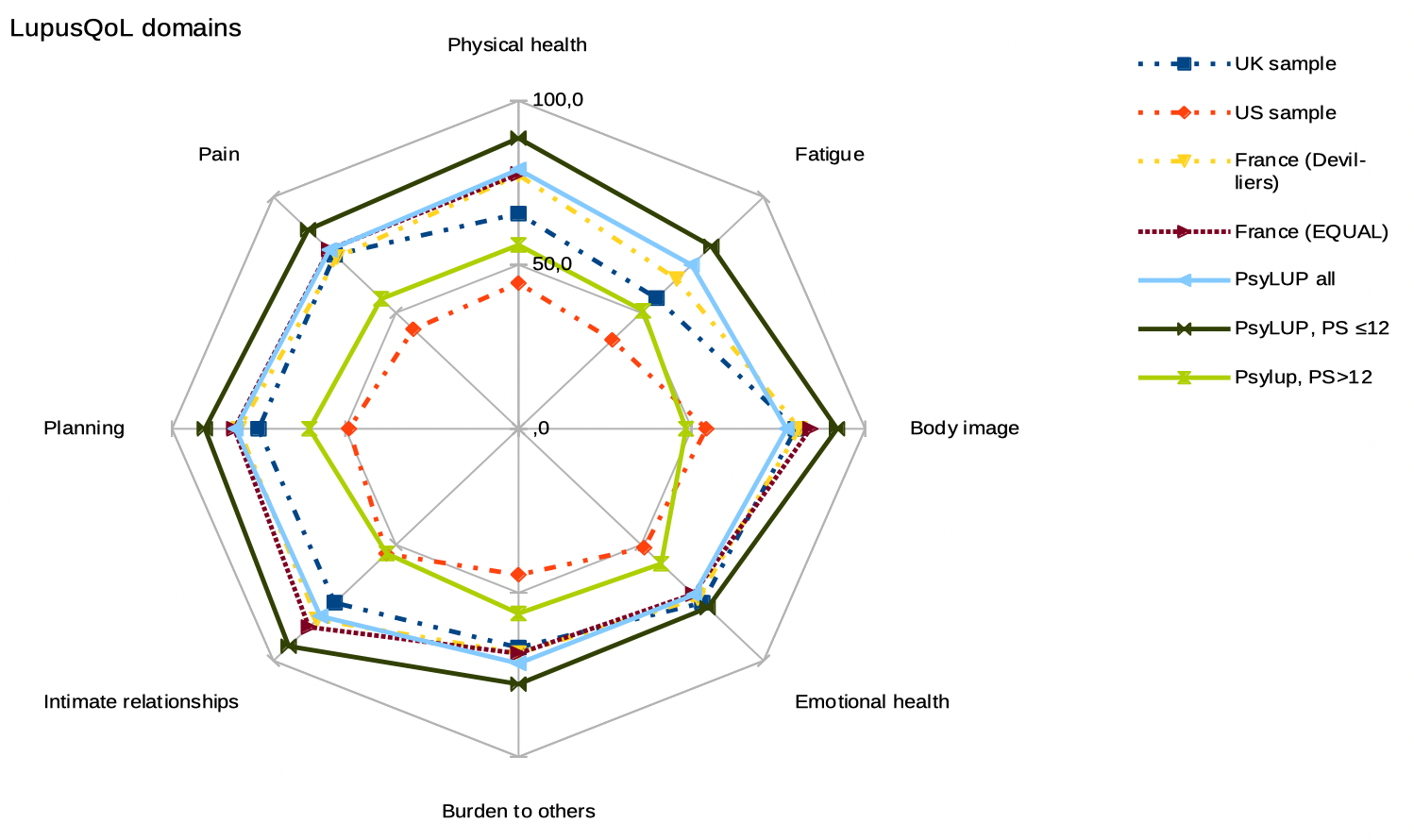
**

Values are shown for all Psy-LUP patients and for patients with normal (P-scale ≤12) or reduced (P-scale >12) social participation.

The axes of the graph represent the different LupusQoL domains, graduated from 0 in the center (worst) to 100 (best). Points represent the mean score of patients from a given cohort in each domain. Lines connect the scores of patients from a same cohort.
